# How important is the N-terminal acetylation of alpha-synuclein for its function and aggregation into amyloids?

**DOI:** 10.3389/fnins.2022.1003997

**Published:** 2022-11-16

**Authors:** Aditya Iyer, Arshdeep Sidhu, Vinod Subramaniam

**Affiliations:** ^1^Department of Biochemistry, Groningen Biomolecular Sciences and Biotechnology Institute, University of Groningen, Groningen, Netherlands; ^2^Nitte University Centre for Science Education and Research, Nitte University (DU), Mangalore, India; ^3^University of Twente, Enschede, Netherlands

**Keywords:** protein aggregation, fibril structure, acetylation, post-translational modifications, alpha-synuclein

## Abstract

N-α-acetylation is a frequently occurring post-translational modification in eukaryotic proteins. It has manifold physiological consequences on the regulation and function of several proteins, with emerging studies suggesting that it is a global regulator of stress responses. For decades, *in vitro* biochemical investigations into the precise role of the intrinsically disordered protein alpha-synuclein (αS) in the etiology of Parkinson’s disease (PD) were performed using non-acetylated αS. The N-terminus of α-synuclein is now unequivocally known to be acetylated *in vivo*, however, there are many aspects of this post-translational modifications that are not understood well. Is *N*-α-acetylation of αS a constitutive modification akin to most cellular proteins, or is it spatio-temporally regulated? Is *N*-α-acetylation of αS relevant to the as yet elusive function of αS? How does the *N*-α-acetylation of αS influence the aggregation of αS into amyloids? Here, we provide an overview of the current knowledge and discuss prevailing hypotheses on the impact of *N*-α-acetylation of αS on its conformational, oligomeric, and fibrillar states. The extent to which *N*-α-acetylation of αS is vital for its function, membrane binding, and aggregation into amyloids is also explored here. We further discuss the overall significance of *N*-α-acetylation of αS for its functional and pathogenic implications in Lewy body formation and synucleinopathies.

## Introduction

**FIGURE 1:**
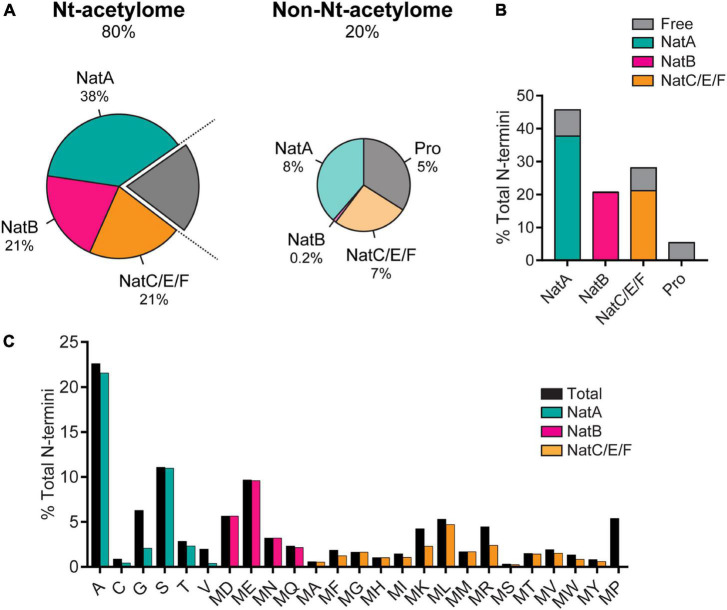
**(A)** The prevalence of N-terminal acetylation in human cells is depicted by separating the proteome into the N-terminal(Nt)-acetylome (80%) and the non-Nt-acetylome (20%). The human Nt-acetylome (the complete set of N-terminal acetylated proteins) was predicted by incorporating experimentally determined Nt-acetylation events (including NatC and NatF related data) to all SwissProt entries (version 57.8) based on the occurrence of the first two amino acids. The chance that a protein will be Nt-acetylated mainly depends on the identity of the first two amino acids. To visualize this concept, the Nt-acetylome can be grouped according to **(B)** NAT substrate class or **(C)** N-terminal amino acid frequency. NatD is not depicted due to its limited coverage. Image reproduced from Ree et al. (2018) licensed under CC BY 4.0. The figure legend is a modified excerpt of the original.

N-terminal acetylation is a post-translational modification carried out by N-terminal acetyltransferases in nascent protein chains during translation (Aksnes et al., 2019). A protein can exist in full, partial and non-acetylated form. N-terminal acetylation involves the addition of an acetyl group to the free alpha-amino group (N-α-group) of the first amino acid in the nascent protein chain by an N-terminal acetyltransferase (Nat) complex (Varland et al., 2015). To date, N-terminal acetylation is considered irreversible because an N-terminal de-acetyltransferase (Ndat) either does not exist in eukaryotic cells or remains to be discovered. We draw a clear distinction between N-terminal acetylation and *N*-α-acetylation in the context of this review. *N*-α-acetylation refers explicitly to the acetylation of the first amino acid (in most cases, methionine). In contrast, N-terminal acetylation may include the acetylation of amino acid residues in the N-terminal region in proteins comprising several amino acids. Protein acetylation also occurs on the ε-amino group of the lysine side chains (*N*-ε-acetylation) catalyzed by a different class of enzymes called lysine acetyltransferases (Choudhary et al., 2014) and on hydroxyl groups of tyrosine/serine/threonine referred to as *O*-acetylation (Yang and Grégoire, 2007). In contrast to Ndats, eukaryotic lysine deacetylases are well-known, and their functions are reviewed elsewhere (Yang and Grégoire, 2007; Choudhary et al., 2014; Xia et al., 2020). In humans, seven Nats have been identified to date — NatA, NatB, NatC, NatD, NatE, NatF, and NatH (Aksnes et al., 2019) — which are responsible for N-terminal acetylation of more than 80% of eukaryotic proteins (Arnesen et al., 2009; Johnson et al., 2010; Aksnes et al., 2016), the rest of the 20% proteome is not known to be acetylated (Ree et al., 2018). Six Nats (NatA to NatF) have broad substrate specificity, except for NatH, which is a dedicated acetylase for actin (Drazic et al., 2018). Each Nat exhibits a strong preference for specific N-terminal residues and (at least) one or two subsequent amino acids required to facilitate N-terminal acetylation (Figure 1).

There is ample evidence in the literature that *N*-α-acetylation of proteins, in general, is an essential mediator of protein function, stability, and localization. N-terminal acetylation impacts protein localization and targeting (Behnia et al., 2004; Setty et al., 2004; Forte et al., 2011; Dikiy and Eliezer, 2014; Park et al., 2015), multi-protein complex formation (Scott et al., 2011; Arnaudo et al., 2013; Monda et al., 2013; Yang et al., 2013; Gao et al., 2016), protein secondary structure (Maltsev et al., 2012; Miotto et al., 2015), protein degradation (Hwang et al., 2010; Shemorry et al., 2013) and aggregation into amyloid fibrils (Kang et al., 2013; Iyer et al., 2016; Watson and Lee, 2019). Of the seven identified Nats, NatB holds particular importance in the context of diseases. NatB acetylates essential proteins at the N-terminus such as tropomyosin, actin, and alpha-synuclein (αS); is required for stability of the actin cytoskeleton; is vital for cell-cycle progression (Starheim et al., 2008), cell proliferation (Ametzazurra et al., 2008); and is implicated in diseases such as hepatocellular carcinoma and Parkinson’s disease (PD) (Polevoda and Sherman, 2003; Ametzazurra et al., 2008; Halliday et al., 2011; Neri et al., 2017).

αS is an intrinsically disordered protein found in high concentrations at the synaptic junctions of neuronal cells. Its precise role in the etiology of PD remains unknown. Several decades of research have not brought us much closer to pinning down its physiological function in eukaryotic cells. αS comprises three domains: the positively charged N-terminal region (aa 1-60) which is involved in membrane binding (Bartels et al., 2010; Lorenzen et al., 2014; Zarbiv et al., 2014; Viennet et al., 2018; Makasewicz et al., 2021), the amyloidogenic NAC domain (aa 61-95) crucial for amyloid formation (Waxman et al., 2009), and the highly charged C-terminal region (aa 96-140), that interacts with polyamines, metal ions, and cellular proteins (Antony et al., 2003; Eliezer, 2013). The observed binding of αS to phospholipid membranes is considered relevant for its function. It is also one of the proposed facilitators of the αS aggregation cascade in addition to point mutations, oxidative stress, truncations, possibly leading to neuronal cell death in PD. Like other eukaryotic proteins, αS is subjected to several post-translational modifications, including phosphorylation, ubiquitination, and acetylation; reviewed elsewhere in detail (Breydo et al., 2012; Barrett and Greenamyre, 2015; Iyer and Claessens, 2019; Zhang et al., 2019). αS is acetylated at the terminal methionine residue (*N*-α-acetylation) by NatB and several lysine residues *in vivo* (*N*-ε-acetylation) by other enzymes, but the physiological impact of acetylation of αS is unclear. We aim to give a critical perspective on the impact of *N*-α-acetylation of αS on its physiological role and pathological aggregation into amyloid fibrils.

## N-terminal acetylation of αS

### A brief history of *N*-α-acetylation

**FIGURE 2:**
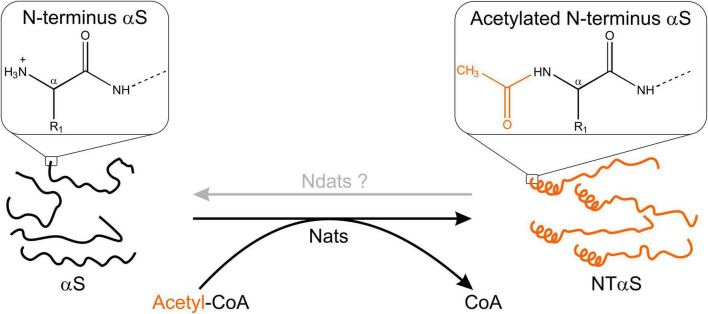
Schematic outline of N-terminal acetylation. N-terminal acetyltransferases (Nats) catalyze the transfer of an acetyl group (CH_3_CO) from acetyl-CoA (Ac-CoA) to the free α-amino group of the protein N-terminus. The transferred acetyl group eliminates a positive charge at the protein N-terminus. In the case of αS, *N*-α-acetylation has been shown to induce helix formation in the first 16 residues (Maltsev et al., 2012). The existence of N-terminal de-acetyltransferases (Ndats) is unknown.

Much before the ongoing debate over its native state, αS was widely accepted as a monomeric, intrinsically disordered protein associated with intracellular membranes and found substantially in a fibrillar state in numerous synucleinopathies. Early investigations into αS focused on the mechanism of aggregation/toxicity and possibly overlooked the role of post-translational modifications occurring in αS. The loss of a positive charge from the N-terminal methionine of αS acetylation affects its secondary structure substantially (Figure 2). *N*-α-acetylation is considered crucial for aggregation of αS into amyloid fibrils (Kang et al., 2012; Yang et al., 2021) and interaction with other binding partners in its native cellular environment (Zabrocki et al., 2008; Runfola et al., 2020). The *N*-α-acetylated form is believed to represent the functional form of the protein, and the debate over its native state being monomeric or tetrameric continues as discussed in the following sections.

**FIGURE 3:**
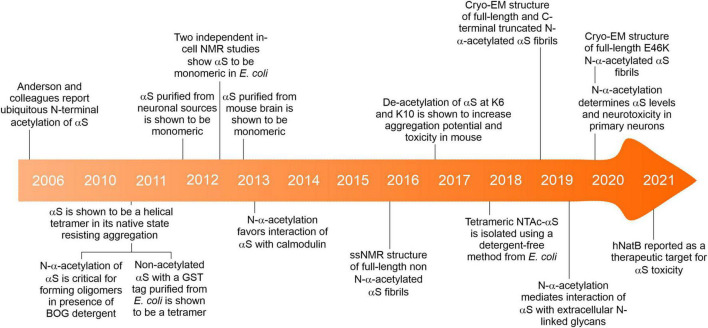
A brief timeline of key events about the *N*-α-acetylation of αS.

The earliest report drawing attention to the presence of *N*-α-acetylation of αS obtained from brain cells and Lewy bodies considered it a passive post-translational modification (Figure 3; Anderson et al., 2006). Before this report, αS was mainly purified and studied from mammalian and non-mammalian sources to ascertain its genetic basis in neurogenerative diseases like PD, multiple system atrophy (MSA), Lewy body dementia, Lewy body variant of Alzheimer’s disease (LBAD), and AD. The relevance of N-α-acetylation of αS gained prominence following a report by the Selkoe group (Bartels et al., 2011), who contradicted the established view of the native state of αS as an intrinsically disordered monomer. Using numerous cell lines and an array of analytical techniques, including EM imaging, circular dichroism spectroscopy, clear-native PAGE (CN-PAGE), and sedimentation-equilibrium analytical ultracentrifugation (SE-AUC), the study reported that native/endogenous αS is an aggregation-resistant helical tetramer in dynamic equilibrium with the monomeric αS species. The study drew parallels to transthyretin amyloidosis, wherein the destabilization of a metastable tetramer in human plasma causes aberrant aggregation of monomers (Quintas et al., 1999).

A widespread debate ensued challenging the tetramer hypothesis in several subsequent studies (Fauvet et al., 2012a,b; Burré et al., 2013), promptly responded to by the primary advocates of the tetramer hypothesis (Bartels and Selkoe, 2013; Dettmer et al., 2013, 2015a,2015b; Luth et al., 2015) and other groups (Ullman et al., 2011; Gurry et al., 2013; Fernández and Lucas, 2018a). The authors’ conclusion that tetrameric αS may dissociate to its monomeric form during cell lysis and widely differing protein purification protocols across research groups gained little reconciliation. The primary authors further showed that the tetrameric species was sensitive to cell-lysis protocols using *in vivo* cross-linking studies that showed the apparent 60-kDa tetramer does not arise from aggregation and that minor 80- and 100-kDa species accompanying varying concentrations of free monomers occurs endogenously in primary neurons as well as neuroblastoma cells that overexpress αS (Dettmer et al., 2013).

Several new questions emerged as a consequence that have been answered in part with ensuing research, while others remain contentious. Could bacterial systems employed to express and purify αS before the Selkoe report (Bartels et al., 2011) not possess the necessary physiological environment for tetramer assembly? Could *N*-α-acetylation of αS *per se* be of enough biophysical consequence to trigger the formation of aggregation-resistant tetramers? These questions were answered, in part, by a report showing that non-acetylated αS purified from *Escherichia coli* (*E. coli*) closely resembled the aforementioned tetrameric species (Wang et al., 2011). However, this construct harbored a 10-residue N-terminal fusion-protein fragment (GPLGSPEFPG) post cleavage of the Glutathione S-transferase (GST) tag that could mimic the biophysical consequences of *N*-α-acetylation of αS. To test if *N*-α-acetylation of αS in bacterial cells could lead to the formation of a tetrameric species, a bacterial co-expression system was used to generate *N*-α-acetylated αS (NTAc-αS). The authors determined that N-terminal acetylation and non-denaturing purification protocols, including the non-ionic detergent octyl β-D-glucopyranoside (BOG), were necessary to observe helical oligomeric αS (Trexler and Rhoades, 2012). In this co-expression system, the NatB acetylase derived from yeast is cloned into a bacterial plasmid, allowing N-terminal acetylation of NatB peptide substrates (MD, ME, MN, MQ; see Figure 1) alongside the overexpression of a target protein (Johnson et al., 2010). The CD spectrum of NTAc-αS showed a helical and presumably tetrameric form when purified in the presence of BOG, while non-acetylated or BOG-free αS was disordered and monomeric. These results implicitly contradicted the hypothesis of a folded αS tetramer in non-acetylation-competent *E. coli* cells used in the previous report (Wang et al., 2011). Could the detergents used during purification protocols lead to the proposed tetrameric state? Using the NatB bacterial co-expression system, Fernández and Lucas, 2018a,b demonstrated a detergent-free method to isolate recombinant tetrameric NTAc-αS. Subsequently, αS was shown to be monomeric by in-cell NMR studies in intact *E. coli* cells (Binolfi et al., 2012; Waudby et al., 2013) and numerous non-neuronal cells (Theillet et al., 2016). The monomer-tetramer debate is far from over but highlights the importance of how subtle environmental changes can cause significant molecular changes in αS. The physiological conditions governing the dynamic equilibrium between monomeric and tetrameric αS remain mysterious. Intuitively, an off-pathway, fibril-resistant αS tetramer can sequester aggregation-competent αS monomers. However, the irreproducibility across labs in isolating the tetrameric species, unknown factors affecting the monomer-tetramer equilibrium and tetramer stability have resulted in reluctant acceptance of its existence.

We speculate that *N*-α-acetylation alone or in combination with other post-translational modifications could be a regulatory step in maintaining an equilibrium between the monomeric and tetrameric states of αS. Tetrameric αS species have been purified from both endogenously expressing and overexpressing mammalian cell lines (Dettmer et al., 2013), ruling out pleiotropic effects of high concentrations. However, in gastrointestinal neuronal cells from rats, the population of tetrameric αS is absent, and these cells constitute primarily monomeric αS (Corbillé et al., 2016). Crowding within mammalian cells alone cannot explain the tetrameric state since in-cell NMR studies in bacterial cytoplasm (Binolfi et al., 2012; Waudby et al., 2013) and the periplasm (McNulty et al., 2006) that are significantly more crowded than mammalian cells (Swaminathan et al., 1997) affirm its monomeric state. Assuming there is an equilibrium between the tetrameric and monomeric species *in vivo*, how are the purified tetrameric αS species stably maintained, preventing their dissociation *in vitro*? A dynamic equilibrium between the tetrameric and monomeric state must be carefully regulated *in vivo*. Long-range interactions between acetyl groups and other amino acids within protein assemblies are well known (Langeberg and Scott, 2015), and transcriptional control via acetylation is one such example (Latham and Dent, 2007). It remains unclear whether *N*-α-acetylation, the purification methodology, the use of detergents, or the choice of a prokaryotic/eukaryotic expression system is crucial for tetramer formation. If *in vivo* cross-linking of tetrameric αS can be achieved, in-cell NMR studies may prove particularly useful in providing concrete evidence of such a species. In addition, how/if the distribution of the monomer-tetramer species depends on cell type and other biochemical factors needs investigation. For instance, glucocerebrosidase 1 deficiency in SH-SY5Y cells has been shown to disfavor the tetrameric αS populations over the monomeric αS population (Kim et al., 2018), while the tetrameric αS population is favored in primary neurons and erythroid cells (Dettmer et al., 2013). Addressing the *N*-α-acetylated state of αS is a promising avenue to probe the existence of a tetrameric species, to understand the possible mechanisms of amyloid formation, and to gain insights into the physiological function of αS.

### Impact of *N*-α-acetylation of αS on biophysical properties and membrane binding

NTAc-αS is suggested to be a physiologically relevant brain species (Bartels et al., 2011; Fauvet et al., 2012b; Burré et al., 2013; Theillet et al., 2016), and several emerging studies have benchmarked its biophysical properties with non-acetylated αS. A summary of all biophysical properties of NTAc-αS is listed in Table 1. Early solution-NMR studies with NTAc-αS revealed that *N*-α-acetylation of αS triggered a helical conformation in the first 16 residues (Maltsev et al., 2012; Dikiy and Eliezer, 2014) *in vitro* and subsequently in live neuronal and non-neuronal cells using in-cell NMR (Theillet et al., 2016). The interactions of non-acetylated αS with membranes have been studied in detail, but interactions with NTAc-αS remain relatively less explored. To the best of our knowledge, membrane binding studies have been carried out only for monomeric NTAc-αS and not for the tetrameric NTAc-αS species. It is well known that the N-terminal region (aa 1–60) of αS is involved in membrane binding. However, emerging studies show that the first 15 residues in αS largely recapitulate the binding properties of full-length αS such as partition constants, molecular mobility, and membrane insertion (Pfefferkorn et al., 2012), and removal of the first 14 residues severely compromises membrane binding (Cholak et al., 2020). How *N*-α-acetylation affects the membrane binding ability of αS is unclear due to conflicting results and differing solvent conditions and membrane compositions used. For example, NTAc-αS showed enhanced membrane binding in two studies (Bartels et al., 2014; Viennet et al., 2018) and no enhancement in other studies (Fauvet et al., 2012a; Maltsev et al., 2012; Iyer et al., 2016).

**TABLE 1:** Effect of N-terminal acetylation on biophysical properties of αS.

Probed parameter		Technique used	Effect of *N*-α -acetylation compared to non-acetylated αS	References
Predominant native state		In-cell NMR	Monomeric	Fauvet et al., 2012a; Theillet et al., 2016
		Mass spectrometry, native-PAGE, CD spectroscopy, sedimentation equilibrium-analytical ultracentrifugation (SE-AUC)	Monomeric	Fauvet et al., 2012a,b; Maltsev et al., 2012; Burré et al., 2013; Iyer et al., 2016
			Tetrameric	Bartels et al., 2011; Wang et al., 2011; Luth et al., 2015; Fernández and Lucas, 2018a,b
Membrane binding of αS monomer		CD spectroscopy, isothermal calorimetry (ITC), nuclear magnetic resonance (NMR)	Enhanced binding to GM1 gangliosides	Bartels et al., 2014
			Comparable binding to GM3, POPS lipids	Maltsev et al., 2012; Bartels et al., 2014; Dikiy and Eliezer, 2014
			Moderately enhanced binding to zwitterionic lipids	Dikiy and Eliezer, 2014; Iyer et al., 2016; O’Leary et al., 2018
Aggregation properties	Amyloid formation rate	ThT fluorescence	Decreased	Kang et al., 2012; Bartels et al., 2014; Gallea et al., 2016; Ruzafa et al., 2017
			No significant effect	Fauvet et al., 2012a; Maltsev et al., 2012; Iyer et al., 2016
			Increased in presence of air-water interface	Viennet et al., 2018
	Heterogeneity in aggregation kinetics	ThT fluorescence	Decreased	Kang et al., 2012; Iyer et al., 2016
Dimer/ Oligomer formation	Oligomer formation	Solid-state nanopores and MD simulations	Decreased	Bu et al., 2017
		SE-AUC	Critically dependent on the presence of BOG detergent	Trexler and Rhoades, 2012
Fibril structure	Fibril height(nm)	Atomic force microscopy (AFM)	No significant effect	Iyer et al., 2016
	Secondary structure	CD spectroscopy	Increased β-sheet content	Iyer et al., 2016; Rossetti et al., 2016
		Raman spectroscopy	Decreased β-sheet content	Watson and Lee, 2019
	Proteinase-K digestion	ThT fluorescence and SDS-PAGE	Increased proteolysis	Iyer et al., 2016; Watson and Lee, 2019
	Periodicity	Scanning transmission electron microscopy (STEM), AFM	Increased no. of monomers per nm of fibril	Iyer et al., 2016

Considering that *N*-α-acetylation leads to loss of a positive charge from the terminal methionine residue, *N*-α-acetylation may affect the interaction between αS and membranes or other binding partners in the cellular milieu. Intuitively, the loss of a positive charge upon *N*-α-acetylation is likely to result in a decreased affinity toward anionic lipid membranes. However, *N*-α-acetylation of αS does not affect its binding to anionic phospholipid membranes with increasing surface charge densities but shows enhanced binding to zwitterionic phospholipid membranes in a curvature-dependent manner (Dikiy and Eliezer, 2014; Iyer et al., 2016; O’Leary et al., 2018). The observation may be reasoned as follows: *N*-α-acetylation of αS increases the propensity of the first 16 residues in the N-terminus to organize into helices (Figure 2). The binding of αS to lipid membranes results in a loss of conformational entropy compensated for by favorable electrostatic interactions and hydrogen bonding. Since NTAc-αS binds with a pre-existing helical conformation, the loss in conformational entropy upon binding to anionic membranes is probably lower for NTAc-αS than for the non-acetylated αS. The lower entropy cost associated with helix formation is balanced by losing the positive charge upon *N*-α-acetylation. The binding of non-acetylated and NTAc-αS to anionic lipid membranes is therefore comparable. In the absence of strong, attractive forces between neutral lipid membranes and αS, the effect of *N*-α-acetylation is likely dominated by the increased propensity of αS to fold into an amphipathic helix. Since the final helical content of both NTAc-αS and non-acetylated αS is comparable, the net free energy gain upon binding of NTAc-αS is higher with neutral lipid membranes resulting in enhanced affinity for NTAc-αS.

Although monomeric non-acetylated αS faithfully mimics NTAc-αS in specific biophysical properties like hydrodynamic radii and conformational change upon binding anionic lipid membranes, it does not reflect the importance of NTAc-αS. *N*-α-acetylation may have yet unknown physiological roles that may not be realized in experiments with purified proteins *in vitro*. For example, a recent study showed that abolishing *N*-α-acetylation of αS led to lower levels of αS and substantially reduced neurotoxicity in substantia nigra of rats (Vinueza-Gavilanes et al., 2020). *N*-α-acetylation of αS may also be possibly prevented *in vivo* by mutating the aspartic acid residue (D) in the second position to a proline residue (P) as recently shown for αS (Vinueza-Gavilanes et al., 2020) and numerous other proteins (Goetze et al., 2009).

Compared to its non-acetylated counterpart, NTAc-αS binds faster to model lipid membranes but forms amyloid aggregates and fibrils slower (Ruzafa et al., 2017; Cholak et al., 2020). However, in the presence of air-water interfaces, the apparent lag-time for NTAc-αS aggregation into amyloid fibrils is nearly twofold lesser than that observed with non-acetylated-αS (Viennet et al., 2018). Further, the presence of the neuronal ganglioside GM1 in model lipid membranes impaired the ability of NTAc-αS to form ThT-positive aggregates (Bartels et al., 2014). Given that the final helical content of both NTAc-αS and non-acetylated αS are comparable, the kinetic barrier for a membrane-bound helical conformation to a β-sheet conformation would also be comparable. If so, why would NTAc-αS aggregate slower on lipid membranes? Perhaps *N*-α-acetylation stabilizes interactions within the helical conformation and orients residues along with the interface such that αS dips further in the membrane, leading to a robust anchoring. While the above-mentioned model lipid membranes provide valuable biochemical insights, the next step must be to validate these observations in mammalian cells. Despite differences in the kinetics of membrane binding, the membrane-bound conformation and the morphology of micelle-induced aggregates of NTAc-αS are invariant with non-acetylated αS. Mimicking the biophysical consequences of N-α-acetylation of αS with or without PD familial mutations, for example, charge swap on terminal methionine, conformational restriction/stabilization of the N-terminal region, are needed to understand the monomer-tetramer equilibrium, aggregation on or in presence of lipid membranes will provide valuable mechanistic and functional insights into the role *N*-α-acetylation of αS.

### How does acetylation of αS impact aggregation in amyloid structures?

The effect of *N*-α-acetylation on the structure of αS monomer and amyloid conformation has been investigated using multiple techniques in recent years. At the monomer level, *N*-α-acetylation does not affect the hydrodynamic radius, electrophoretic properties, and oligomerization potential of αS, suggesting minimal changes in the overall structure and biochemistry as compared to non-acetylated αS (Fauvet et al., 2012a; Kang et al., 2012; Gallea et al., 2016; Ni et al., 2019). However, NMR studies using ^1^H-^15^N HSQC show a significant difference in the chemical environment of the first nine residues and increased helical propensity of the first 12 residues (Fauvet et al., 2012a; Kang et al., 2012; Maltsev et al., 2012). The increased helicity of the N-terminus on acetylation mirrors the structural transitions observed in αS in the presence of model membranes, albeit only in a small region of the protein (Davidson et al., 1998; Eliezer et al., 2001; Georgieva et al., 2008). The acetyl carbonyl (C=O) group can participate in a hydrogen bond with the amino H (N-H) group from subsequent amino acids, which can stabilize a helix by sealing its fraying end (Fairman et al., 1989; Chakrabartty et al., 1993; Doig et al., 1994). NTAc-αS with helical N-terminus may facilitate its transition from a random coil to an α-helix *in vivo*, on interaction with a membrane surface, due to lower entropic cost and favorable dipole interactions associated with adding residues to an α-helix rather than initiating the helix (Zimm and Bragg, 1959; Creighton, 1993). In addition to the N-terminus, weak long-range interactions around residues 28–31, 43–46, 50, and 50–66 were also reported in acetylated αS (Kang et al., 2012). All these sites, toward the end of the N-terminal region (aa 1–60) and the beginning of the NAC region (aa 61–95) of αS, are associated with αS function and familial forms of PD (Polymeropoulos et al., 1997; Krüger et al., 1998; Zarranz et al., 2004; Lesage et al., 2013; Proukakis et al., 2013; Pasanen et al., 2014).

Histidine-50 is one of the copper (I) binding sites of αS that is mutated in the familial form of PD (H50Q mutation) (Sung et al., 2006; Proukakis et al., 2013). Non-acetylated αS binds copper via a coordination complex involving the N-terminal amine group of methionine-1, backbone and side chain of aspartate-2, and the imidazole ring of histidine-50 (Dudzik et al., 2011). A clear difference in methionine-1 and aspartate-2 environment on acetylation in NMR studies (Fauvet et al., 2012a; Kang et al., 2012) is predictive of different αS-copper interaction in acetylated and non-acetylated form. H50Q mutation in non-acetylated αS increases the aggregation of monomeric αS into amyloid structures, with minor changes in the secondary structure and negligible effect on the overall copper binding capacity (Uversky et al., 2001; Ghosh et al., 2013). Copper binding in *N*-α-acetylated H50Q protein (the *in vivo* form of H50Q mutation) is impaired, likely due to a double hit at the copper coordination complex; lack of the N-terminal amine, and the absence of the imidazole side chain at position 50 (Mason et al., 2016). Loss of copper-binding in acetylated H50Q is likely to interfere with the proposed ferrireductase activity of αS, leading to defects in metal homeostasis *in vivo* (Davies et al., 2011; Mezzaroba et al., 2019).

*N*-α-acetylated αS, like non-acetylated αS, aggregates into oligomers and fibrils under various experimental conditions (Kang et al., 2012; Maltsev et al., 2012; Gallea et al., 2016; Lima et al., 2019; Watson and Lee, 2019). There are varying reports for the effect of acetylation on both oligomers and fibrils. The extent of oligomerization of acetylated αS has been reported to be the same (Fauvet et al., 2012a; Kang et al., 2012) as well as reduced (Bu et al., 2017). Further, acetylated oligomers and fibrils show morphological and spectral features similar to unmodified αS, except for increased β-sheet and helical content in acetylated αS oligomers (Gallea et al., 2016; Iyer et al., 2016). The acetylated forms of familial PD mutants, E46K, H50Q, and A53T, show increased aggregation in 3,4-dihydroxyphenylacetaldehyde (DOPAL), dopamine, and SDS micelles, in comparison to wild-type acetylated αS (Ruzafa et al., 2017; Lima et al., 2019). Changes in fibrillization kinetics of wild-type αS upon acetylation are also ambiguous. Some studies report no significant difference (Fauvet et al., 2012a; Maltsev et al., 2012; Iyer et al., 2016), while others report slower kinetics, especially in the elongation rate (Kang et al., 2012; Ruzafa et al., 2017; Watson and Lee, 2019). This reduced elongation rate could be due to a helical secondary structure at the N-terminus that likely hinders the conversion of a monomer into the typical fibrillar β-sheet conformation (Kang et al., 2012). Acetylated αS is reported to yield distinct polymorphs (Watson and Lee, 2019) with likely increased structural homogeneity within a population (Iyer et al., 2016). The increased structural homogeneity in a fibril population may arise from a monomeric pool that is “structurally homogenous” (Figure 2). *N*-α-acetylation of αS results in a homogenous ensemble wherein 16 amino acids are in a helical conformation, leading to the nucleation of a homogenous population of fibrils. In a distinct polymorph, the reduced elongation rate can also be due to lower Thioflavin-T sensitivity toward acetylated αS, as Thioflavin-T fluorescence assay is sensitive to changes in topological features (Sidhu et al., 2018; Watson and Lee, 2019).

**FIGURE 4:**
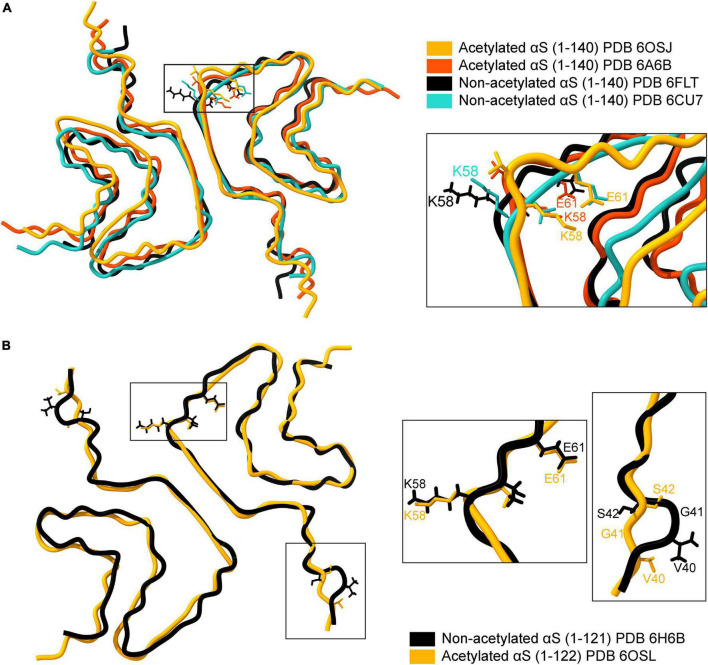
Comparison of available cryo-EM structures of the full-length acetylated (yellow, PDB ID: 6OSJ and orange, PDB ID:6A6B) and non-acetylated αS fibrils (black, PDB ID: 6FLT and blue, PDB ID:6CU7). **(A)** The backbone overlay of acetylated and non-acetylated αS fibrils is shown. The residue K58 in both acetylated αS fibrils is flipped inward, forming a salt bridge between K58-E61 (see inset). In contrast, the salt bridge is broken in acetylated αS fibrils due to the outward flip of K58. **(B)** The backbone overlay generated using ChimeraX of truncated (1-121/2) acetylated (yellow, PDB ID: 6OSL) and non-acetylated αS fibrils (black, PDB ID: 6H6B) depicting flipped K58 residues irrespective of acetylation state and minor loop fluctuations around the G41 residue.

Structurally, fibrils formed by acetylated and non-acetylated αS show a mix of similar and distinct features. An overlay of four full-length αS structures, two with acetylation and two without acetylation, reveal an analogous backbone arrangement (Figure 4A; Tuttle et al., 2016; Li B. et al., 2018; Li Y. et al., 2018; Ni et al., 2019). Both types of fibril structures are formed of two protofilaments that intertwine in a twisted fibril morphology along a 21 screw axis – placing two monomers ∼180° to each other with an interaction surface in the center (Li B. et al., 2018; Li Y. et al., 2018; Ni et al., 2019). The N-α-acetylated protofilaments show a left-handed helical twist of –0.72° and a rise of ∼4.8 Å (Li Y. et al., 2018; Ni et al., 2019), while the non-acetylated protofilaments show a right-handed helical twist of 179.1° and a rise of 2.4 Å (Li B. et al., 2018). The dimer interaction surface in acetylated fibrils is formed by a hydrophobic steric zipper between residues histidine-50 to glutamate-57. Additionally, electrostatic interactions between histidine-50 and lysine-45 from one monomer and glutamate-57 from another monomer, and salt bridges between lysine-58: glutamate-61 (K58-E61) and glutamate-46: lysine-80 (E46-K80) stabilize the fibril core (Li Y. et al., 2018; Ni et al., 2019). In non-acetylated fibrils, the steric zipper is formed by residues further in the NAC region. Residues 55–62 are disordered (ssNMR studies) or do not form the steric zipper (cryoEM studies). The dimer interaction surface is formed by valine-71 to valine-82 in ssNMR studies and by glycine-68 to alanine-78 in cryoEM structures. Moreover, lysine-58 is flipped outward, resulting in the absence of the K58-E61 salt bridge (Figure 4A; Tuttle et al., 2016; Li Y. et al., 2018).

The stabilizing effect of the salt-bridges on protein structure is well known, particularly in the case of αS. The compromised salt bridge between E46 and K80 side chains in an E46K variant of αS leads to a structurally homogenous yet entirely different fibril structure (consisting of one fibril species) and is more pathogenic compared to the wild-type αS (Boyer et al., 2020). A summary of all the available fibril structures of αS in PDB is listed in Table 2, with corresponding indicators for the K58-E61 salt bridge in each structure. An exciting facet of the αS fibril structure emerges concerning the K58-E61 salt bridge. Full-length *N*-α-acetylated αS fibrils have the K58-E61 salt bridge intact in sharp contrast to non-*N*-α-acetylated αS fibrils. The presence of the K58-E61 salt bridge is not influenced by *N*-α-acetylation alone but also by C-terminal truncation, phosphorylation of Tyr39, E46K mutation, and fibril polymorphism. The stark differences in the orientation of K58 cannot be an artifact of differing aggregation conditions since, in a single study employing identical aggregation conditions, the K58-E61 salt bridge was preserved in full-length *N*-α-acetylated αS fibrils and 1–103 *N*-α-acetylated αS fibrils but broken in 1–122 *N*-α-acetylated αS fibrils (Ni et al., 2019). Further, comparing structures of C-terminal truncated 1–121/2 αS fibrils suggests little or no role of *N*-α-acetylation on the orientation of K58 (Figure 4B). Why is the orientation of K58 sensitive to *N*-α-acetylation in full-length αS fibrils but not in C-terminal truncated αS fibrils? Further experiments elucidating the driving force for the K58-E61 salt bridge could be exciting and may give us a better understanding of salt-bridges in the stability of αS fibrils. It is unclear if the orientation of K58 and the salt bridge between K58-E61 is physiologically relevant to its cellular function or pathological aggregation of αS. The outward orientation of K58 may render non-acetylated, and C-terminally truncated fibrils exposed to ubiquitination or SUMOylation (signal for proteasome-induced degradation) or acetylation by lysine acetylases.

**TABLE 2:** Comparison of available αS fibril structures and an overview of K58-E61 salt bridge.

		Orientation				
N-terminal-αS	Salt bridge K58-E61	K58	E61	PDB ID	Method	References
Full-length						
NH2-αS, 1–140	Broken	Out	In	2N0A	ssNMR	Tuttle et al., 2016
*N*-α-acetyl-αS, 1–140	Present	In	In	6A6B	cryoEM	Li Y. et al., 2018
*N*-α-acetyl-αS, 1–140	Present	In	In	6OSJ	cryoEM	Ni et al., 2019
NH2-αS, 1–140	Broken	Out	In	6FLT	cryoEM	Guerrero-Ferreira et al., 2018
Polymorph						
NH2-αS rod	Broken	Out	In	6CU7	cryoEM	Li B. et al., 2018
NH2-αS twister	Broken	Out	In	6CU8	cryoEM	Li B. et al., 2018
NH2-αS fibril polymorph 2A	Broken	Out	Out	6SSX	cryoEM	Guerrero-Ferreira et al., 2019
NH2-αS fibril polymorph 2B	Broken	Out	Out	6SST	cryoEM	Guerrero-Ferreira et al., 2019
*N-α-acetyl-αS filament: MSA Type I	Broken	Out	In	6XYO	cryoEM	Schweighauser et al., 2020
*N-α-acetyl-αS filament: MSA Type II-1	Broken	Out	In	6XYP	cryoEM	Schweighauser et al., 2020
*N-α-acetyl-αS filament: MSA II-2	Broken	Out	In	6XYQ	cryoEM	Schweighauser et al., 2020
Truncations/Modifications						
NH2-αS,1-121	Broken	Out	In	6H6B	cryoEM	Guerrero-Ferreira et al., 2018
*N*-α-acetyl-αS, 1–103	Present	In	In	6OSM	cryoEM	Ni et al., 2019
*N*-α-acetyl-αS, 1–122	Broken	Out	In	6OSL	cryoEM	Ni et al., 2019
NH2-αS phosphoTyr39 (twist dimer)	Broken	Out	Out	6L1T	cryoEM	Zhao et al., 2020b
NH2-αS phosphoTyr39 (twist trimer)	Broken	Out	Out	6L1U	cryoEM	Zhao et al., 2020b
PD mutants						
NH2-αS E46K, 1–140	Broken	Out	Out	6UFR	cryoEM	Boyer et al., 2020
*N*-α-acetyl-αS E46K, 1–140	Broken	Out	Out	6L4S	cryoEM	Zhao et al., 2020a
*N*-α-acetyl-αS A53T, 1–140	Present	In	In	6LRQ	cryoEM	Sun et al., 2020
NH2-αS H50Q Wide Fibril	Present	In	In	6PES	cryoEM	Boyer et al., 2019
NH2-αS H50Q Narrow Fibril	Present	In	In	6PEO	cryoEM	Boyer et al., 2019

N-terminal de-acetyltransferases (Ndats) are not known in eukaryotic cells as yet, suggesting constitutional *N*-α-acetylation of αS by N-terminal acetyltransferases (Nats). Could it be possible that Nats decline in function or decrease in expression levels in an age-dependent manner? Such a scenario would result in a decrease in *N*-α-acetylated αS over time and possibly affect its function and interaction with its binding partners. It has been shown by several groups that *N*-α-acetylated αS fibrils are less cytotoxic compared to non-*N*-α-acetylated αS fibrils. Studies investigating the absolute amounts of *N*-α-acetylated αS and non-*N*-α-acetylated αS in healthy and diseased patients would be a significant step forward. The proposed hypothesis on Nats draws parallels from a study investigating the effect of the NAD-dependent deacetylase sirtuin 2 (SIRT2) on the aggregation potential and cytotoxicity of αS. The authors showed that lysine residues acetylated at the ε-amino positions in the N-terminal region of αS (K6 and K10) from mice brains could be deacetylated by SIRT2. The deacetylation event exacerbated its aggregation potential and toxicity *in vitro* and in the substantia nigra of rats (de Oliveira et al., 2017). Furthermore, mutating K6 and K10 residues to create αS variants that are acetylation-resistant or mimic constitutive acetylation showed that acetylation at these residues prevents αS aggregation in the substantia nigra of rats. The remarkable changes in aggregation potential and toxicity of αS *in vivo* resulting from acetylation of N-terminal lysine residues are intriguing. The authors proposed a model in which the age-dependent increase of SIRT2 in the brain, with the concomitant decrease of acetylated αS, leads to increased αS aggregation and the worsening of the expected defects in the autophagy-lysosome pathway (ALP) associated with aging.

The wild-type interactions of αS protofibrils are perturbed in familial PD mutations. The observation is not surprising as most of the mutations associated with the familial form of PD (H50Q, G51D, A53T, A53E) are located at the dimerization interface. The H50Q mutation disrupts the H50-K45-E57 interaction, while the E46K mutation breaks the E46-K80 salt bridge (Li B. et al., 2018). In A53T mutations, the dimerization core is formed by only two residues, Tyr-59 and Lys-60, instead of seven residues (H50-E57) in wild-type αS (Sun et al., 2020). Thus, these mutations can be expected to weaken the fibril core, resulting in morphological differences and greater fragmentation that consequently may increase seeding potential (Zhao et al., 2020a).

In structural studies, acetylated and non-acetylated αS fibrils could seed aggregation reactions and were cytotoxic (Tuttle et al., 2016; Li B. et al., 2018; Li Y. et al., 2018). In wild-type αS, acetylated αS seeds faithfully template fibril morphology across multiple seeding reactions, while non-acetylated αS seeds show poorer templating (Watson and Lee, 2019). Since the seed molecule’s conformation is critical in templating reactions, an unstable fibril core in non-acetylated αS, due to the absence of the K58-E61 salt bridge, may lead to poor templating (Sidhu et al., 2016). NMR studies show that in seeded aggregations of acetylated αS monomers with fibril seeds and off-pathway oligomers, the first 11 residues interact with the seeds in both the cases—successful templating with fibril seeds and unsuccessful templating with off-pathway oligomers. The observation suggests that the N-terminal interaction of acetylated αS is the first point of contact between a seed and a free monomer, irrespective of templating outcome (Yang et al., 2021). The differences between oligomers and fibrils from acetylated and non-acetylated αS monomers are likely due to the acetyl group. Still, some of the differences, at least, could also be due to differences in fibril preparation protocols used in each study. Differences in protein concentration; solution conditions like buffer, salt, metal ions, small molecules; agitation; incubation time have a significant effect on the kinetics and morphology of αS fibrils (Hoyer et al., 2002; Heise et al., 2005; Powers and Powers, 2006; Vilar et al., 2008; Knowles et al., 2009; Morel et al., 2010; Bousset et al., 2013; Buell et al., 2013, 2014; Sidhu et al., 2014; Buell, 2019; Panuganti and Roy, 2020). Since all the studies compared here have differences in the parameters mentioned above, a direct comparison to arrive at an empirical conclusion is challenging.

### Effect of N-terminal acetylation on the physiological function of αS

More than 300 post-translational modifications (PTMs) are known to occur in proteins (Clark et al., 2005), but a handful of these are known for αS, and their implications have been discussed in detail (Beyer, 2006; Zhang et al., 2019). These modifications include acetylation, phosphorylation, nitration, glycosylation, SUMOylation, ubiquitination, di-tyrosine crosslinking, and methionine oxidation. While the impact of PTMs in αS has been studied extensively in isolation, very few studies have considered the impact of *N*-α-acetylation in concert with the modifications mentioned above. Experiments in yeast show that deletion of NatB selectively increased localization of αS to cytoplasm and not plasma membrane as in wild-type yeast (Zabrocki et al., 2008). Evidence for the role of N-terminal acetylation of αS in its function are scarce and are still emerging. Since *in vivo* αS is universally present in the acetylated form (Bartels et al., 2011; Fauvet et al., 2012b; Burré et al., 2013; Theillet et al., 2016), all the studies with endogenous αS represent functions of acetylated αS. However, most of the studies with recombinant αS report behavior of non-acetylated αS. Only systematic comparative studies of αS behavior from endogenous and recombinant αS can delineate the effects of N-terminal acetylation. Limited studies that focus on the acetylated αS show that acetylated forms are involved in Lewy body associated pathology, metal homeostasis and synaptic function. Mass-spectrometry based studies from postmortem tissue of dementia with Lewy bodies (DLB) and PD patients, show full-length and truncated acetylated αS forms (Ac-αS_1–139_, Ac-αS_1–119_ Ac-αS_1–103_) and no non-acetylated forms, suggesting that in both disease and healthy conditions acetylation is present (Öhrfelt et al., 2011). This is consistent with another study that identified multiple truncated acetylated forms (Ac- αS_1–6_, Ac- αS_13–21_, Ac- αS_35–43_, Ac- αS_46–58_, Ac- αS_61–80_, Ac- αS_81–96_, Ac- αS_103–119_) of αS in Lewy body enriched fractions of PD patient samples (Bhattacharjee et al., 2019). In addition to brain tissues, only NTAc-αS can be detected in blood from Alzheimer’s patients and not the non-acetylated form, which is an indicator of neuronal death (Pero-Gascon et al., 2020). These studies highlight the importance to study physiologically relevant biochemistry of αS in acetylated forms to find better inhibitors for αS aggregation and to identify biomarkers.

αS is a copper binding protein with two sites for interaction with copper: Met 1-Met 5 and Ala 49-His 50 (Dudzik et al., 2011). Copper binding at Met 1–Met 5 is different for acetylated and non-acetylated αS forms. Copper binding in non-acetylated form at position Met 1–Met 5 results in a redox active state that can reduce metals while acetylated αS, though binds Cu^2+^, does not exhibit redox behavior (Garza-Lombó et al., 2018). The copper binding behavior of αS at the N-terminus is observed both in solution and membrane bound conformations (Dudzik et al., 2013). Since both N-terminal acetylation and copper binding increase the propensity of αS to adopt α-helical conformation, it is likely that they synergistically contribute to αS interaction with synaptic vesicles.

#### Could N-terminal acetylation of αS be a priming event?

Post-translational modifications can be reversible or irreversible, and the regulatory dynamics of these modifications may give vital insights into protein function. Unlike reversible PTMs, like phosphorylation, glycosylation, ubiquitination, SUMOylation, methionine oxidation, nitration that may be rapidly added or removed from a protein under varied metabolic or pathologic cues, N-α-acetylation has been thought to be irreversible and occurring co-translationally. However, there is emerging evidence that acetylation of N-termini of proteins does necessarily occur co-translationally (Dormeyer et al., 2007). When ^15^N isotope-enriched non-acetylated αS was delivered into A2780, HeLa, RCSN-3, B65, and SK-N-SH cells using electroporation and was found to be *N*-α-acetylated entirely within 5 h (Theillet et al., 2016). These evidences suggest that cells prefer *N*-α-acetylated αS. It may be energetically more favorable for ubiquitous acetylation of αS to occur co-translationally.

There is no evidence of the existence of N-terminal de-acetyltransferases (Ndats), suggesting the irreversible nature of *N*-α-acetylation. This observation opens new avenues to investigate the existence of N-terminal de-acetyltransferases (Ndats) and other regulatory mechanisms that could (dys)regulate *N*-α-acetylation of αS. NMR studies have shown that *N*-α-acetylation induces stable α-helix formation in the first 16 amino acid residues in αS (Maltsev et al., 2012). *N*-α-acetylation of αS occurs co-translationally in eukaryotes and therefore precedes all other PTMs. Not surprisingly, the various permutations of PTMs mentioned above in αS preparations have consistently reported *N*-α-acetylation of αS at the least. We speculate that *N*-α-acetylation may “prime” αS for subsequent PTMs vital to its function and explain the cellular need to acetylate the N-terminus co-translationally. Our speculation is based on several observations: (a) *N*-α-acetylation led to plasma membrane localization of acetylated αS in yeast while non-acetylated αS remained in the cytoplasm. Further, the study showed decreased levels of Ser129 phosphorylation in non-acetylated αS compared to acetylated αS (Zabrocki et al., 2008). (b) Crosstalk between acetylation and other PTMs in a given protein is well known in eukaryotes (Yang and Seto, 2008) and impacts cell fate, and has implications for aging (Ree et al., 2018). For example, acetylation of histone H3 at K9/27 positions crosstalk with phosphorylation at S10/28 positions, respectively, to affect downstream gene expression (Latham and Dent, 2007). (c) The formation of a stabilized helix upon *N*-α-acetylation may provide lysine acetylases a helical scaffold (compared to disordered chain in non-acetylated αS) to effectively acetylate lysine residues in the 6th and 10th position in αS. Such scaffolds are well known in the context of signaling proteins and multi-protein complexes in eukaryotes (Langeberg and Scott, 2015). (d) The lack of Ndats potentially highlights the importance of *N*-α-acetylation of αS, with as yet unknown modes of regulation. Typically, modifications closely involved in regulatory processes are reversible processes (Martin, 2007). Examples of such reversible processes include protein (de)phosphorylation, (de)acetylation, (de)adenylylation, and (de)ADP-ribosylation. Additionally, Acetyl-CoA is a key metabolite in cellular metabolism and its consumption for the *N*-α-acetylation of αS indicates a necessary protein modification. (e) *N*-α-acetylation has been shown to inhibit protein targeting to the endoplasmic reticulum (Forte et al., 2011).

The priming role of *N*-α-acetylation of αS suggested here may have evaded sight as it likely does not require genomic regulation or quantitative changes in αS levels. Thus, *in vivo* studies investigating the impact of *N*-α-acetylation of αS on subsequent PTMs, especially phosphorylation, may help us understand if *N*-α-acetylation has a priming function. Understanding the crosstalk between *N*-α-acetylation and S129 phosphorylation is vital since several reports show accelerated inclusion formation and cellular toxicity in different models triggered by S129 phosphorylation (Smith et al., 2005; Sugeno et al., 2008). Additionally, more than 90% of αS deposited in Lewy bodies (LBs) in PD patients is phosphorylated at S129 while healthy individuals exhibit roughly 4% S129 phosphorylation (Arawaka et al., 2017).

## Directions for future research

The physiologically native state of αS is unquestionably *N*-α-acetylated. The observation has been determined exhaustively in numerous mammalian cells and organisms. It remains irrefutably an irreversible modification in αS so far. The impact of *N*-α-acetylation of αS in the context of pathological consequences (aggregation into toxic oligomers, fibrils, and higher-ordered aggregates) is increasingly being investigated. With the advent of cryo-EM, we are beginning to see structural details of αS fibrils at unprecedented spatial resolutions. Emerging studies are benchmarking the fibril structure of NTAc-αS housing PD familial mutations with endogenous αS fibrils isolated from diseased patients. However, despite these achievements, the impact of *N*-α-acetylation on the function of αS is still murky.

It is vital to understand how acetylation imbalance in αS manifests *in vivo* and which physiological consequences of the imbalance lead to neurotoxicity (de Oliveira et al., 2017). In this respect, a detailed proteomics study documenting the ratio of acetylated and non-acetylated αS over the progress of Lewy body formation would be remarkable. Emerging studies have shown enough evidence of *N*-α-acetylation affecting several downstream processes in living cells. A recent study demonstrated that *N*-α-acetylation of αS determines αS levels and subsequent toxicity in primary neurons (Vinueza-Gavilanes et al., 2020). Using point mutants that altered or blocked *N*-α-acetylation, the authors demonstrated that blocking *N*-α-acetylation led to a decrease in αS levels in live primary neurons and concomitantly reduced neurotoxicity. The prospect of blocking *N*-α-acetylation of αS by NatB is exciting, yet, maybe challenging for drug discovery strategies given that NatB acetylates ∼20% of cellular proteins. CRISPR-based strategies in the future may be able to edit the first two N-terminal amino acids and demonstrate if *in vivo* blocking *N*-α-acetylation of αS may help to decrease αS levels. Although NTAc-αS is recognized as the physiologically relevant species in healthy brain cells – in both the soluble and insoluble fractions of brain tissues of PD patients (Anderson et al., 2006) – the use of non-acetylated αS is rampant in emerging literature. The use of NTAc-αS must be encouraged, and NTAc-αS should be a gold standard for all studies investigating this multi-faceted protein (Lashuel et al., 2013) concerning conformational changes, oligomerization and aggregation propensities, lipid interactions, and other cellular binding partners.

In the future, we must focus our efforts toward elucidating (a) the effect of co-occurring *N*-α-acetylation and other PTMs in αS on its membrane (un)binding, oligomer/fibril structure, and corresponding aggregation kinetics, (b) the effect of co-occurring *N*-α-acetylation and familial PD mutations on αS function and aggregation into fibrillar structures, (c) the relation between the level of *N*-α-acetylation of αS and the progression rate of neurodegeneration in synucleinopathies, (d) the relation between metal-ion (dys)homeostasis and cellular models of synucleinopathies wherein levels of *N*-α-acetylation can be modulated, and (e) the complex relation between aggregation rates, diffusion coefficients, macromolecular crowding and *N*-α-acetylated αS *in vivo*. We may also want to investigate the interplay of regulatory factors (sirtuins) or genetic circuits triggered in PD patients and *N*-α-acetylation levels. While the physiological function of αS remains evasive, *N*-α-acetylation of αS presents an exciting path for future research.

## Author contributions

AI, AS, and VS wrote the manuscript. All authors contributed to the article and approved the submitted version.
